# *Helicobacter pylori *and cancer among adults in Uganda

**DOI:** 10.1186/1750-9378-1-5

**Published:** 2006-11-07

**Authors:** Robert Newton, John L Ziegler, Delphine Casabonne, Lucy Carpenter, Benjamin D Gold, Marilyn Owens, Valerie Beral, Edward Mbidde, D Maxwell Parkin, Henry Wabinga, Sam Mbulaiteye, Harold Jaffe, the Uganda Kaposi's Sarcoma Study Group

**Affiliations:** 1 Epidemiology and Genetics Unit, Dept. of Health Sciences, First Floor, Seebohm Rowntree Building, University of York, Heslington, York, YO10 5DD, UK; 2 Uganda Cancer Institute and Makerere University Medical School, Kampala, Uganda; 3 Cancer Research UK Epidemiology Unit, University of Oxford, Richard Doll Building, Roosevelt Drive, Oxford, OX3 7LF, UK; 4 MRC Programme on AIDS, Uganda Virus Research Institute, PO Box 49, Entebbe, Uganda; 5 Division of Pediatric Gastroenterology and Nutrition, Department, Pediatrics, Emory University School of Medicine, 2040 Ridgewood Dr., NE, Atlanta, GA 30322, USA; 6 International Agency for Research on Cancer, 150 Cours Albert Thomas, Lyon, France; 7 Centers for Disease Control and Prevention, 1600 Clifton Road, Atlanta, Georgia, 30333, USA; 8 Dept. of Veterans Affairs and the University of California, San Francisco, to the International Agency for Research on Cancer, France; 9 Dept. of Public Health, Oxford University, to the MRC Programme on AIDS, Entebbe, Uganda

## Abstract

Data from Africa on infection with *Helicobacter pylori *(*H. pylori*) are sparse. Therefore, as part of an epidemiological study of cancer in Uganda, we investigated the prevalence and determinants of antibodies against *H. pylori *among 854 people with different cancer types and benign tumours. Patients were recruited from hospitals in Kampala, Uganda, interviewed about various demographic and lifestyle factors and tested for antibodies against *H. pylori*. In all patients combined, excluding those with stomach cancer (which has been associated with *H. pylori *infection), the prevalence of antibodies was 87% (723/833) overall, but declined with increasing age (p = 0.02) and was lower among people who were HIV seropositive compared to seronegative (p < 0.001). Otherwise, there were few consistent epidemiological associations. Among those with stomach cancer, 18/21 (86%) had anti-*H. pylori *antibodies (odds ratio 0.8, 95% confidence intervals 0.2–2.9, p = 0.7; estimated using all other patients as controls, with adjustment for age, sex and HIV serostatus). No other cancer site or type was significantly associated with anti-*H. pylori *antibodies. The prevalence of *H. pylori *reported here is broadly in accord with results from other developing countries, although the determinants of infection and its' role in the aetiology of gastric cancer in Uganda remain unclear.

## Background

The work described in this report was part of an epidemiological study of cancer in Kampala, Uganda [1-7]. Data from Africa on infection with *Helicobacter pylori *(*H. pylori*) are sparse. Therefore, we examine here the role of antibodies against *H. pylori *in relation to the risk of cancer and investigate the prevalence and determinants of infection among 854 people with different cancer types and benign tumours.

## Materials and methods

Full details of the methods are provided elsewhere [1,2]. Briefly between 1994 and 1998, we recruited adults 15 years or older with a new diagnosis of cancer from the wards and out-patient clinics of the main hospitals in Kampala, Uganda. After informed consent and counselling, patients were interviewed and tested for infection with HIV-1 using the Cambridge Bioscience Recombigen ELISA (Cambridge, MA) on sera or the GACELISA method (Murex, Dartford, UK) on saliva. Cancer diagnoses were established by histology or other laboratory investigation, where possible. Diagnoses made on clinical grounds alone were reviewed by the investigators. The study was approved by the Committee on Human Research (VA Medical Centre and University of California San Francisco) and by the Uganda National Council for Science and Technology.

Following HIV testing, remaining sera were stored at minus 80 Celsius and were later shipped on dry ice to the Centres for Disease Control and Prevention, Atlanta, USA, for *H. pylori *testing. Assays were performed by a single investigator who was blind to the diagnosis of the patient from whom the blood was obtained. Briefly, *H. pylori *organisms were grown overnight in brucella broth (GIBCO Laboratories, Madison, WS) with 10% fetal bovine serum (Sigma, St. Louis, MO), 5 μg/ml trimethoprim and 10 μg/ml vancomycin (Sigma). *H. pylori *antigen extraction and protein isolation were done by gentle freeze-thaw sonication (Heat System, Farmingdale, NY) [8,9]. A standard protein assay (Pierce, Rockford, IL) was used to determine the accurate and reproducible quantity of solid-phase antigen for our microtitre research ELISA [10]. Cross-reactivity and specificity of *H. pylori *whole-cell antigens has been described previously [9,10]. Optical density (OD) values at a wavelength of 492 nm were determined in triplicate for each biopsy-confirmed control patient sera, using a standard 96-well microtiter plate ELISA spectrophotometer (Fisher Scientific, Pittsburgh, PA). The mean OD values were then calculated. The ELISA cut-off values were derived using known *H. pylori*-positive and negative control sera as previously described [9,11]. In previous validation studies the assay has demonstrated a high and reproducible sensitivity and specificity in African patients as compared to upper endoscopy and biopsy; sensitivity >88%, specificity >90% [9,11].

Serological results were available for 50 people with non-malignant manifestations of HIV disease, recruited from the out-patient department of Mulago hospital and for 804 patients with cancer or benign tumours, for whom a stored blood sample was available for testing. The latter group comprised people with cancers of the oral cavity (26), oesophagus (38), stomach (21), liver (52), skin (22), breast (69), cervix (190), ovary (22), prostate (10), penis (14), eye (63), and non-Hodgkin's lymphoma (46), Hodgkin's disease (24), Kaposi's sarcoma (46), other cancer sites or types (126) and benign tumours (35).

Data were computerised by trained clerks using EPI-INFO software (CDC, Atlanta) and statistical analyses were conducted using STATA (STATA Corporation, Texas). Only a small proportion of those tested were seronegative for antibodies against *H. pylori *(optical density <0.9) or had an indeterminate result (optical density 0.9–1.3). In all analyses, those with indeterminate results were considered to be seronegative. In order to examine potential confounding factors, the risk of being seropositive for antibodies against *H. pylori *was examined in relation to various social and demographic factors among all patients combined (but excluding stomach cancer, which has been associated with *H. pylori *infection). Odds ratios (OR) were estimated using unconditional logistic regression modelling with adjustment for sex, age group (<30, 30–45, 46+) and HIV serostatus. When calculating odds ratios in relation to anti-*H. pylori *antibodies, for each cancer site or type, the comparison group included all other patients with the exception of stomach cancer. Tests for association used the χ^2 ^test for linear trend on one degree of freedom and all p values are 2-sided. Risk factors for high titres of antibodies against *H. pylori *were examined amongst all patients combined (excluding stomach cancer), but no clear associations were identified and the data are not shown.

## Results

*H. pylori *antibody status was available for 854 people; 87% (741) were seropositive, 4% (38) were seronegative and 9% (75) had an indeterminate result. Table 1 shows the association between *H. pylori *serostatus and selected social and demographic factors among all patients excluding those with stomach cancer. The prevalence of antibodies did not vary with sex, but declined with increasing age (χ^2^_1 _= 5.1, p = 0.02) and was lower among people who were HIV seropositive compared to seronegative (χ^2^_1 _= 16.2, p < 0.001). No other factor examined was associated with antibodies against *H. pylori *with the exception of religion: the prevalence of antibodies was higher among Muslims than among Christians (χ^2^_1 _= 5.6, p = 0.02).

**Table 1:** Odds ratios (OR) for *H. pylori *seropositivity according to various social and demographic factors among all patients (excluding those with stomach cancer)

**Variable**	**Number positive/total**	**OR (95% CI)**^1^	**Variable**	**Number positive/total**	**OR (95% CI)**^1^
**Sex**			**Time to market**		
Male	247/284	1.0	<30 minutes	323/375	1.0
Female	476/549	0.9 (0.6–1.3)	30+ minutes	278/320	1.0 (0.7–1.6)
		χ^2^_1 _= 0.5, p = 0.5			χ^2^_1 _= 0.0, p = 0.9
**Age group**			**Size of community**		
<36 years	269/309	1.0	>100 houses	220/262	1.0
36–50 years	250/282	1.0 (0.6–1.6)	10–99 houses	421/482	1.3 (0.9–2.1)
51+ years	204/242	0.5 (0.3–0.9)	<10 houses	51/57	1.6 (0.6–4.1)
		χ^2^_1 _= 5.1, p = 0.02			χ^2^_1 _= 1.0, p = 0.3
**HIV serostatus**			**Ever travel from home**		
Negative	514/574	1.0			
Positive	201/250	0.4 (0.3–0.6)	Yes	91/109	1.0
		χ^2^_1 _= 16.2, p<0.001	No	599/688	1.4 (0.7–2.5)
					χ^2^_1 _= 1.0, p = 0.3
**Region of residence**			**Household size**		
Kampala	161/188	1.0	<6 people	357/410	1.0
Rest of Uganda	560/643	1.0 (0.6–1.6)	6+ people	354/411	0.8 (0.5–1.2)
		χ^2^_1 _= 0.0, p = 0.9			χ^2^_1 _= 1.6, p = 0.2
**Tribe**			**Number of siblings**		
Baganda	349/410	1.0	<6 siblings	379/438	1.0
Other	374/423	1.2 (0.8–1.8)	6+ siblings	333/384	0.9 (0.6–1.4)
		χ^2^_1 _= 0.8, p = 0.4			χ^2^_1 _= 0.1, p = 0.8
**Religion**			**Number of children**		
Muslim	95/100	1.0	<7 children	341/397	1.0
Christian	619/723	0.3 (0.1–0.8)	7+ children	290/329	1.1 (0.7–1.8)
		χ^2^_1 _= 5.6, p = 0.02			χ^2^_1 _= 0.1, p = 0.8
**Occupation**			**Tobacco consumption**		
Cultivator	334/387	1.0			
Other	382/439	1.1 (0.7–1.9)	Never smoker	562/647	1.0
		χ^2^_1 _= 0.1, p = 0.8	Past smoker	81/92	1.2 (0.6–2.5)
			Current smoker	68/82	0.7 (0.4–1.4)
					χ^2^_1 _= 0.5, p = 0.5
**Education level**			**Alcohol consumption**		
No school	153/175	1.0			
Primary	361/415	0.9 (0.5–1.6)	Never	391/443	1.0
Secondary/tertiary	191/225	0.7 (0.4–1.4)	About once/week	134/157	0.8 (0.5–1.4)
		χ^2^_1 _= 1.0, p = 0.3	2–4 days/week	91/106	0.9 (0.5–1.6)
			Most days	93/113	0.7 (0.4–1.2)
					χ^2^_1 _= 1.6, p = 0.2
**Household Income (Ug. Sh.)**			**Lifetime number of sexual partners**		
15,000+	464/533	1.0	1–2 partners	182/204	1.0
<15,000	196/230	0.9 (0.6–1.5)	3–9 partners	305/352	0.9 (0.5–1.5)
		χ^2^_1 _= 0.1, p = 0.8	10+ partners	196/232	0.7 (0.4–1.3)
					χ^2^_1 _= 1.2, p = 0.3

1. Odds ratios adjusted for age group (<30, 30–45, 46+), sex and HIV serostatus

Table 2 shows the association between anti-*H. pylori *antibodies and specific cancer sites or types, together with the proportion of cancers with a laboratory verification of diagnosis. Overall, 62% of cancers were diagnosed on the basis of histology or other laboratory investigation, but the figure varied by cancer site or type, being lowest for prostate cancer (44%) and highest for Kaposi's sarcoma (91%). Of those people with stomach cancer, 90% (19/21) had the diagnosis confirmed by a laboratory investigation.

**Table 2:** *H. pylori *serostatus for different cancer sites or types and non-malignant conditions, together with the percentage of each cancer with laboratory verification of diagnosis

**Cancer site or type**	**Percentage with laboratory verification of diagnosis**	**Number anti-*H. pylori *antibody positive/total**	**Odds ratio (95% CI)^1^**	**χ^2^_1 _and p value**
**Stomach**	90%	18/21	0.8 (0.2–2.9)	χ^2^_1 _= 0.1, p = 0.7
**All controls^2^**	-	723/833	1.0	-
**Oral**	56%	21/26	0.6 (0.2–1.7)	χ^2^_1 _= 0.9, p = 0.4
**Oesophagus**	45%	37/38	5.1 (0.7–38)	χ^2^_1 _= 2.5, p = 0.1
**Liver**	56%	44/52	0.7 (0.3–1.6)	χ^2^_1 _= 0.8, p = 0.4
**Skin**	77%	18/22	0.6 (0.2–1.9)	χ^2^_1 _= 0.8, p = 0.4
**Breast**	62%	62/69	1.4 (0.6–3.5)	χ^2^_1 _= 0.6, p = 0.4
**Cervix**	50%	173/190	1.6 (0.9–2.8)	χ^2^_1 _= 2.1, p = 0.2
**Ovary**	73%	20/22	1.1 (0.3–5.0)	χ^2^_1 _= 0.0, p = 0.9
**Prostate**	44%	7/10	0.3 (0.1–1.4)	χ^2^_1 _= 2.4, p = 0.1
**Penis**	57%	12/14	0.9 (0.2–4.3)	χ^2^_1 _= 0.0, p = 0.9
**Conjunctiva**	66%	33/38	1.3 (0.5–3.5)	χ^2^_1 _= 0.3, p = 0.6
**Other eye**	52%	22/25	1.1 (0.3–3.9)	χ^2^_1 _= 0.0, p = 0.9
**Non-Hodgkin's lymphoma**	76%	39/46	0.8 (0.3–1.8)	χ^2^_1 _= 0.4, p = 0.5
**Hodgkin's lymphoma**	83%	20/24	0.6 (0.2–1.9)	χ^2^_1 _= 0.7, p = 0.4
**Kaposi's sarcoma**	91%	36/46	0.7 (0.3–1.6)	χ^2^_1 _= 0.9, p = 0.4

1. Odds ratios adjusted for age group (<30, 30–45, 46+), sex and HIV serostatus

2. The comparison group for the calculation of odds ratios includes all other cancers and non-malignant conditions, excluding stomach cancer

Note: H. Pylori serostatus: negative – optical density (O.D.) 0.0–0.8; indeterminate – O.D. 0.9–1.3; positive – O.D. 1.4–4.0. For the purposes of this analysis, those with an indeterminate result were considered to be seronegative.

Among 21 cases with stomach cancer, one was HIV seropositive, 13 were women, one was aged <30 years, three were aged between 30–45 years and 17 were aged 46+ years. Among those with stomach cancer, 86% (18/21) were seropositive for antibodies against *H. pylori *antigens, compared to 87% (761/871) of the comparison group (odds ratio = 0.8, 95% confidence intervals 0.2–2.9; χ^2^_1 _= 0.1, p = 0.7). Nor was there a statistically significant association between anti-*H pylori *antibodies and any other cancer site or type examined.

## Discussion

Here we report the first data from Uganda on the seroprevalence of antibodies against *H. pylori*. The prevalence of 87% was broadly comparable to that reported from other hospital series elsewhere on the African continent: 79% in Algeria [12], 71% in Côte d'Ivoire [12], 79% in the Democratic Republic of Congo [13], 85% in Nigeria [14] and 86–93% in South African blacks [15,16]. In the only other study of *H. pylori *from Uganda, Wabinga [17] identified a high frequency of colonisation in gastric endoscopic biopsies from people with upper gastrointestinal symptoms.

Data on the determinants of infection with *H. pylori *in Africa are scant. The prevalence of infection has been found to rise through childhood, reaching over 70% in early adulthood [15] and has been associated with markers of Hepatitis A infection [15], premastication of infant's food [18] and low social class in some studies, but not others (reviewed in reference [19]). In this study, few consistent associations between either the prevalence or titre of anti-*H. pylori *antibodies and any of the risk factors examined were identified. The reasons for the lower prevalence identified in this study among people aged over 50 years and among HIV infected people are unclear. Similarly, since no consistent differences between religious groups have been identified to date in this study [1-7], it is likely that the differences in the prevalence of anti-*H. pylori *antibodies observed here, between Christians and Muslims, arose by chance.

It is recognised that serum antibodies against *H. pylori *may decline because of the development of gastric changes, such as malignancy, that can suppress or kill the infection [20]. Case-control studies have therefore shown inconsistent associations between antibodies against *H. pylori *and gastric cancer (reviewed in reference [21]). The lack of an association in this study and in the only other from Africa in which serum antibodies were measured [22] is, therefore, unsurprising. The lack of statistical power in this study (based on only 21 cases of stomach cancer) and the incomplete diagnostic verification may have further reduced the ability to detect an association. Indeed, the only study from Africa to find an association between gastric cancer and *H. pylori *involved assessment of infection status microscopically in tissue taken from areas adjacent to disease and included only six people with the tumour [23].

The apparent increase in the incidence of gastric cancer seen in Uganda since the 1960s [24], though not statistically significant, may have been influenced by improvements in diagnosis and is at odds with the decline seen throughout much of the rest of the world. The role of *H. pylori *and other factors in the aetiology of gastric cancer in Uganda and elsewhere in Africa remains unclear.
